# The mechanism of PFK-1 in the occurrence and development of bladder cancer by regulating ZEB1 lactylation

**DOI:** 10.1186/s12894-024-01444-5

**Published:** 2024-03-13

**Authors:** Rong Wang, Fei Xu, Zhengjia Yang, Jian Cao, Liqi Hu, Yangyang She

**Affiliations:** Department of Urology, Hangzhou Linping TCM Hospital, No.101 Yuncheng Street, Tangxi Town, Linping District, Hangzhou City, 311106 China

**Keywords:** Bladder cancer, PFK-1, ZEB1, Lactylation, Glycolysis

## Abstract

**Background:**

Bladder cancer (BC) is one of the most common malignancies of the genitourinary system. Phosphofructokinase 1 (PFK-1) is one of member of PFK, which plays an important role in reprogramming cancer metabolism, such as lactylation modification. Zinc finger E-box-binding homeobox 1 (ZEB1) has been demonstrated to be a oncogene in many cancers. Therefore, this study was performed to explore the effects of PFK-1 on the lactylation of ZEB1 in BC development.

**Methods:**

Cell viability was measured using the CCK-8 kit. The glucose assay kit and lactate assay kit were used to detect glucose utilization and lactate production. The DNA was purified and quantified by qRT-PCR.

**Results:**

In the present study, we found that ZEB1 expression levels were significantly elevated in bladder cancer cells. Impaired PFK-1 expression inhibits proliferation, migration, and invasion of BC cells and suppresses tumour growth in vivo. We subsequently found that knockdown of PFK-1 decreases glycolysis, including reduced glucose consumption, lactate production and total extracellular acidification rate (ECAR). Mechanistically, PFK-1 inhibits histone lactylation of bladder cancer cells, and thus inhibits the transcription activity of ZEB1.

**Conclusion:**

Our results suggest that PFK-1 can inhibit the malignant phenotype of bladder cancer cells by mediating the lactylation of ZEB1. These findings suggested PFK-1 to be a new potential target for bladder cancer therapy.

**Supplementary Information:**

The online version contains supplementary material available at 10.1186/s12894-024-01444-5.

## Introduction

Bladder cancer (BC) is one of the most common malignancies of the genitourinary system [1]. With the increasing incidence and mortality, bladder cancer causes more than 170,000 deaths each year. When it invades the muscle, BC develops into two distinct sub-types: non-muscle invasive BC (75%) and muscle invasive BC (25%), with muscle invasive BC having a higher risk of metastasis. Despite significant advances in clinical treatments such as surgery, chemotherapy and radiotherapy over the past decades, the 5-year survival rate for patients with BC remains low [2]. Lymph node metastasis is considered to be the main route of metastasis and the main cause of poor prognosis in BC, with the 5-year survival rate of patients decreasing from 77.6 to 18.6% [3]. Lack of early diagnosis and effective treatment leads to poor clinical regression in BC patients, thus, it is essential to explore novel molecular mechanisms and therapeutic targets for BC [4].

Phosphofructokinase (PFK) is the second rate-limiting enzyme in glucose metabolism, catalysing the conversion of fructose 6-phosphate to fructose 1, 6-bisphosphate. PFK consists of 2 isoforms, PFK-1 and PFK-2, which catalyse the conversion of glucose to 1, 6-bisphosphofructose and 2, 6-bisphosphofructose, respectively, consuming 1 molecule of ATP [5]. PFK-1 exists in the organism as a tetramer, but the composition of the tetramer varies in different organs. PFK1 is thought to play an important role in reprogramming cancer metabolism [6, 7]. In normal cells, PFK-1 is inhibited by high levels of ATP, phosphoenolpyruvic acid (PEP) and citrate, and activated by adenosine phosphate, PFK-2 and others. However, in malignant tumors there are several regulatory mechanisms that can increase PFK expression, such as the activation of proto-oncogenes such as ras and c-ras, which can enhance the expression of PFK-1. PFK-1 catalyses the conversion of fructose 6-phosphate to fructose 1,6-bisphosphate, which induces the tetramerisation of pyruvate kinase (PK) into the glycolytic enzyme complex, thereby promoting the glycolytic pathway. It was found that clotrimazole can directly inhibit the activity of PFK and alter the structure of 6-phosphofructokinase-1 and its affinity for substrate. However, the role of PFK-1 in BC and the mechanism by which it inhibits glucose metabolism are unknown and require further study. It has been reported that TP53-induced regulators of glycolysis and apoptosis can inhibit glycolysis by reducing the expression of PFK-1 [8]. Martinez-Outschoorn et al. reported that the human breast cancer cell line MCF-7, which highly expresses TIGAR, can be resistant to estrogenic drugs [9].

Zinc finger E-box-binding homeobox 1 (ZEB1), as an important transcription regulatory factor in the epithelial-mesenchymal transition (EMT) process, can participate in the EMT process by binding to the E-box site [10]. Many studies demonstrate that inhibiting the ZEB1 expression can suppress the cancer progression [11, 12], including BC [13]. Recently, histone lactylation was newly discovered by Zhang et al. In BC progression, lactylation modification has been demonstrated to regulate the LCN2 to participate in the BC progression [14]. However, whether lactylation modification can regulate the expression of ZEB1 and affect the progression of BC has not been reported yet. High levels of PFK-1 promotes the glycolysis development, inducing the production lactate, which may lead to the aggravate the occurrence of lactylation modification. Therefore, we speculate that PFK-1 may regulate the expression of ZEB and participate in the progression of BC through lactylation modification.

Here in the current study, the aim was to investigate the role and mechanism of PFK-1 in the progression of BC. We found that impaired PFK-1 can affect the level of glycolysis and slow down the growth of BC cells in vitro and in vivo. We then found that damaged PFK-1 could inhibit lactylation of ZEB1, thereby inhibiting BC progression.

## Materials and methods

### Cell culture and treatment

Human ureteral epithelial cells SV-HUC-1, BC cell lines including UM-UC-1, UM-UC-3, RT4, RT112 and T24 were purchased from Procell Life Sciences (Wuhan, China). SV-HUC-1 cells were cultured in Ham’s F-12 K medium (Macgene, China) supplemented with 10% fetal bovine serum (FBS) (Gibco, MA, USA). UM-UC-1, UM-UC-3, RT4, RT112 and T24 cells were cultured in RPMI-1640 medium (Gibco, MA, USA) supplemented with 10% FBS. All cells were cultured in 5% CO_2_ at 37 °C.

The si-PFK-1 and PFK-1 overexppressing plasmids and their negative control were synthesized by Gemma Shanghai. UM-UC-1 and RT112 cells were grown at 90% fusion. Transfection was performed using Lipofectamine 3000 regent according to the instructions. The culture medium was changed 6 h after transfection, and PCR was performed to detect transfection efficiency at 24–48 h after transfection.

### Reverse transcription-quantitative polymerase chain reaction (RT-qPCR)

In line with the supplier’s directions, the TRIzol reagent (Invitrogen, USA) was used for isolation of total RNA from BC cells. Following reverse transcription by the RevertAid™ cDNA Synthesis kit (Takara, Japan), cDNA samples were subjected to RT-qPCR analysis in ABI 7500 Real-Time PCR system (ABI, USA) by means of the SYBR Green Supermix kit (Bio-Rad, USA). The sequences of adopted primers were listed: PFK-1, forward: 5′-AGCGTTTCGATGATGCTTCAG-3′ and reverse: 5’-GGAGTCGTCCTTCTCGTTCC-3’; ZEB1, forward: 5′- TTACACCTTTGCATACAGAACCC-3′ and reverse: 5’-TTTACGATTACACCCAGACTGC-3’;β-actin, forward: 5′-AGGGAAATCGTGCGTGAC-3′ and reverse, 5′-CGCTCATTGCCGATAGTG-3′. The relative expression was calculated with the 2–ΔΔCt method and presented by normalization to β-actin.

### Cell counting kit-8 (CCK-8)

Cell viability was measured using the CCK-8 kit (Dojindo, China). Cells (5 × 10^3^ cells/well) were cultured for 5 days and 10 µL CCK-8 reagent was added to each well. After 2 h of incubation, the absorbance was measured at 450 nm using a multi-function micro-plate reader (BioTek, USA).

### Colony formation

BC cells were suspended to the concentration of 1.0 × 10^3^/ml. Then, 3 ml transfected cells were inoculated into 6-well plates and incubated at 37 °C for 14 days, then each well was washed with 1×PBS, followed by fixing in methanol for 20 min. Finally, the colonies were stained with crystal violet for 25 min. Photographs were then taken to assess colony formation. Image J software was employed to assay the number of colonies.

### Transwell

For cell migration detection, 1 × 10^3^ Cells in 200 µL serum-free medium were inoculated into the upper wells and 600 µL medium supplemented with 10% FBS was added to the lower wells. After 48 h, the cells in the upper layer were removed and the cells in the lower layer were fixed with ethanol and stained with 0.1% crystal violet for 30 min. For cell invasion detection, the cells were seeded onto the Transwells pre-coated with 100 µL of Matrigel. Other operations are consistent with cell migration detection. Photographs were taken and the number of migrated cells was evaluated using Image J software.

### Detect of glucose and lactate content

The glucose assay kit (Jiancheng, China) and lactate assay kit (Jiancheng, China) were used to detect glucose utilization and lactate production, respectively, according to the manufacturer’s instructions. Briefly, Cells were seeded into 6-well plates for 12 h. Then, the culture media were replaced with fresh complete medium and the cells were incubated for additional 6 h. The media were then collected for determination of glucose and lactate content. The relative glucose and lactate content were assessed ground on the standard curve and normalized to protein content using the BCA Protein assay (Jiancheng, China) .

### Extracellular acidification rate assay (ECAR)

BC cells (50,000 cells/well) were plated in Seahorse XF96 plates and treated with LPS in the presence or absence of LA or 2-DG for Seahorse assay to detect the extracellular acidification rate (ECAR) and oxygen consumption rate (OCR) by using ECAR determination kit (Agilent Technologies, Santa Clara, CA, USA) and OCR determination kit (Agilent Technologies,) respectively. For glycolysis stress test, cells were starved in glucose-free media containing treatments for 1 h. ECAR and OCR was measured prior to and after sequential addition of glucose, oligomycin and 2-DG every 5 min. For MitoStress test, cells were incubated in glucose-containing media with treatments for 1 h. measurements were performed prior to and after sequential addition of oligomycin, FCCP and Rotenone/Antimycin A every 5 min. Finally, the Wave software was applied to analyze data.

### Western blot

Total proteins were isolated using RIPA buffer (Beyotime, China). Protein concentration was then evaluated using a bicinchoninic acid (BCA) kit (Beyotime, China). Proteins (20 µg/lane) were separated by 10% SDS-PAGE and then transferred to a polyvinylidene difluoride (PVDF) membrane. After blocking with skimmed milk (5% concentration), the primary antibodies and secondary antibodies (Abcam, USA) were applied sequentially to the membrane. TBST was used to wash the membrane. Finally, proteins were identified by chemiluminescence (Bio-Rad, USA).

### Immunoprecipitation (IP)

UM-UC-1 or RT112 cells were harvested, 100 µL Cell IP Lysis Buffer (containing protease inhibitors) was added. Then the cells are lysed on ice or at 4 °C for 30 min, centrifuged at 12,000 g for 30 min, and the supernatant is extracted. Next, the lysates were pretreated with 50 µL of protein A/G immune magnetic beads (Bimake, Houston, TX, USA) and were subjected to immunoprecipitation with antibodies obtained, including anti-lactyl-histone H3 (Lys18) rabbit mAb (anti-H3K18la, Life Technologies, CA, USA) and l-lactyllysine (PTM Biolabs, Chicago, IL, USA). The results were analyzed by western blot.

### Chromatin immunoprecipitation (ChIP) assay

For protein-DNA cross-linking, BC cells were fixed with 1% formaldehyde (F8775, Sigma) for 10 min at room temperature. The collected cells were then incubated on ice with DTT solutions for 10 min. Cell nuclei were extracted through centrifugation at 2000×g for 5 min. The chromatin lysates were immunoprecipitated with specific antibodies for H3K18la (PTM Biolabs) or the negative control IgG (Millipore, Billerica, MA, USA) at 4 °C overnight. The chromatin-antibody complex was obtained by pulling down with Protein G Magnetic beads (Cell Signaling Technology, CA, USA). Next, chromatin fragments were eluted from beads, and the protein-DNA cross-linking was reversed by proteinase K incubation (Cell Signaling Technology). Finally, the DNA was purified and quantified by qRT-PCR.

### Luciferase assay

ZEB1 promoter region was cloned into pGL3-basic vector (Promega, Madison, WI, USA) and then co-transfected into BC cells together with sh-PFK-1 and lactic acid (LA). Forty-four hours after transfection with Lipofectamine 3000 (Invitrogen), cells were harvested for measurement of the firefly luciferase activity or the Renilla activity (the internal control) on a Dual-Luciferase® Reporter Assay System (Promega).

### BC mouse model establishment

Female BALB/c nude mice (8-week-old; 20 ± 2.9 g) were supplied by Vital River Experimental Animal Technology Co., Ltd (Beijing, China). Mice were fed with food and water ad libitum and were fed at specific pathogen-free conditions at 20˚C, 60% humidity and alternating 12-h light/dark cycles. A total of 12 athymic nude mice were included in sh-NC (*n* = 6) and sh-PFK-1 (*n* = 6) groups. The UM-UC-1 cells suspended in 200 µL PBS (1 × 10^6^) were inoculated subcutaneously under the armpit of the nude mice. When the tumor volume reached 50 mm^3^, the short and long diameter of the tumor and the weight of mice were measured every week. 4 weeks later, the mice were sacrificed using intraperitoneal injection of 4% pentobarbital (160 mg/kg). The sodium tumor tissues were haversted and weighed. All animal experiments were previously approved by the Maidekangna Animal Science.

### Statistical analysis

Data processing was performed with GraphPad Prism version 8.0 (GraphPad software, USA). Data are expressed as mean ± standard deviation. Student’s *t*-test or ANOVA was used to compare data among tow or multiple groups, respectively. *P* < 0.05 was considered significant difference.

## Result

### Knockdown of PFK-1 inhibited BC progression

**Fig. 1:**
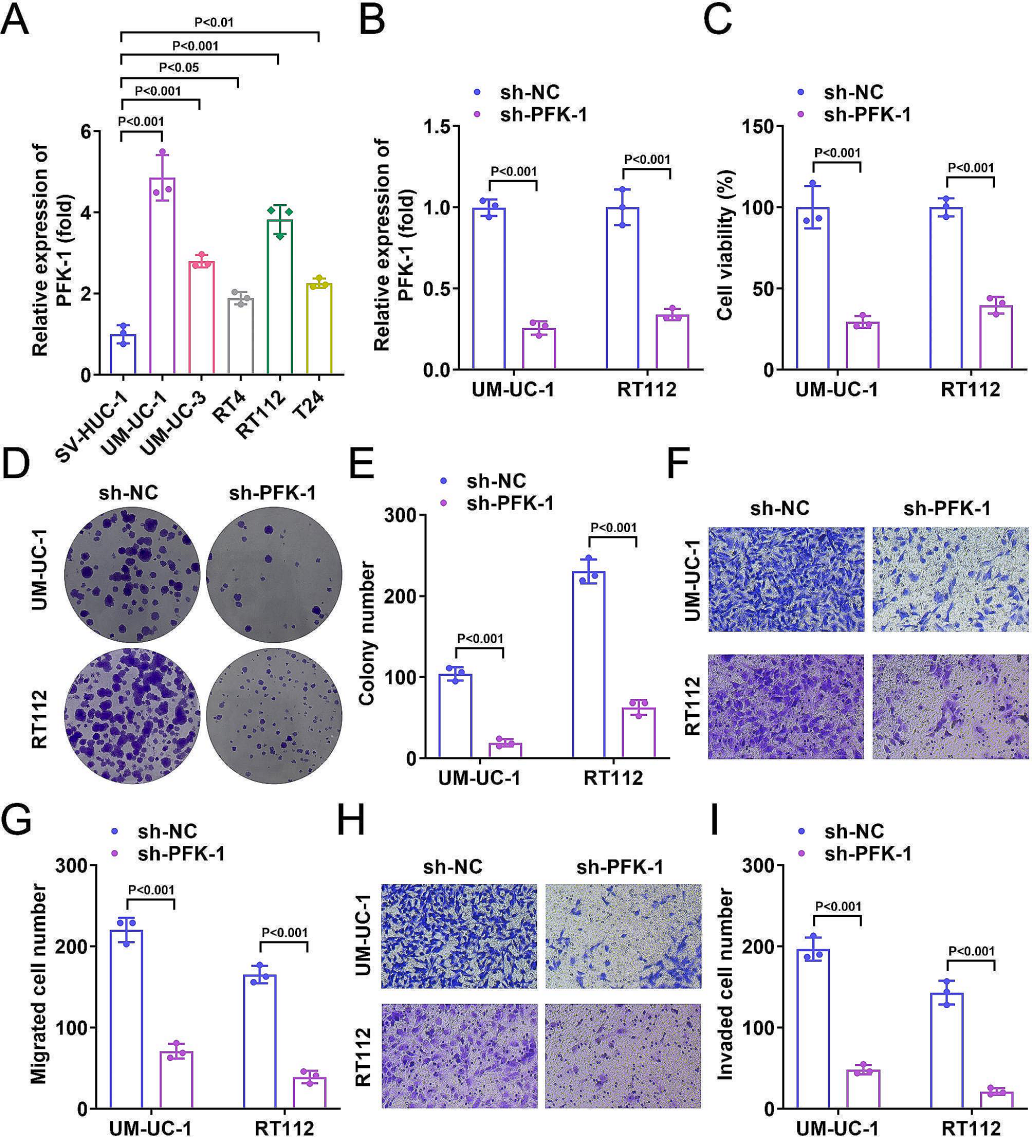
PFK-1 silencing inhibited BC progression. (**A**) The mRNA levels of PFK-1 were increased in SV-HUC-1, UM-UC-1, UM-UC-3, RT4, RT112, and T24 cells. UM-UC-1 and RT112 cells were used for next experiments. (**B**) After sh-PFK-1 transfection, the mRNA levels of PFK-1 in UM-UC-1 and RT112 cells was decreased. After sh-PFK-1 transfection, (**C**) CCK-8 assay indicated that cell viability of UM-UC-1 and RT112 cells was decreased. (**D**-**E**) Colony formation assay showed the cell growth of UM-UC-1 and RT112 cells were decreased. Transwell assay showed migration (**F**-**G**) and invasion (**H**-**I**) of UM-UC-1 and RT112 cells was decreased

We first analyzed PFK-1 expression in normal ureteral epithelial cells and various BC cell lines to investigate the role of PFK-1 in BC. The results showed that PFK-1 was significantly increased in UM-UC-1, UM-UC-3, RT4, RT112, and T24 cell lines compared to SV-UC-1 cell lines (Fig. 1A). The PFK-1 levels were the highest in UM-UC-1 and RT112 cells. So, UM-UC-1 and RT112 cells were used for next experiments. To determine the effect of PFK-1 in BC, we constructed the sh-PFK-1 to down-regulate PFK-1 expression in BC cell lines (Fig. 1B). We found that cell viability was reduced in both UM-UC-1 and RT112 transfected with sh-PFK-1 compared to the sh-NC group (Fig. 1C). Moreover, PFK-1 knockdown inhibited proliferation (Fig. 1D, E), migration (Fig. 1F, G) and invasion (Fig. 1H, I) of UM-UC-1 and RT112 cells. These results suggest that impaired PFK-1 expression may inhibit cell growth through multiple biological functions of the BC cells.

### Knockdown of PFK-1 inhibited glycolysis of BC cells

**Fig. 2:**
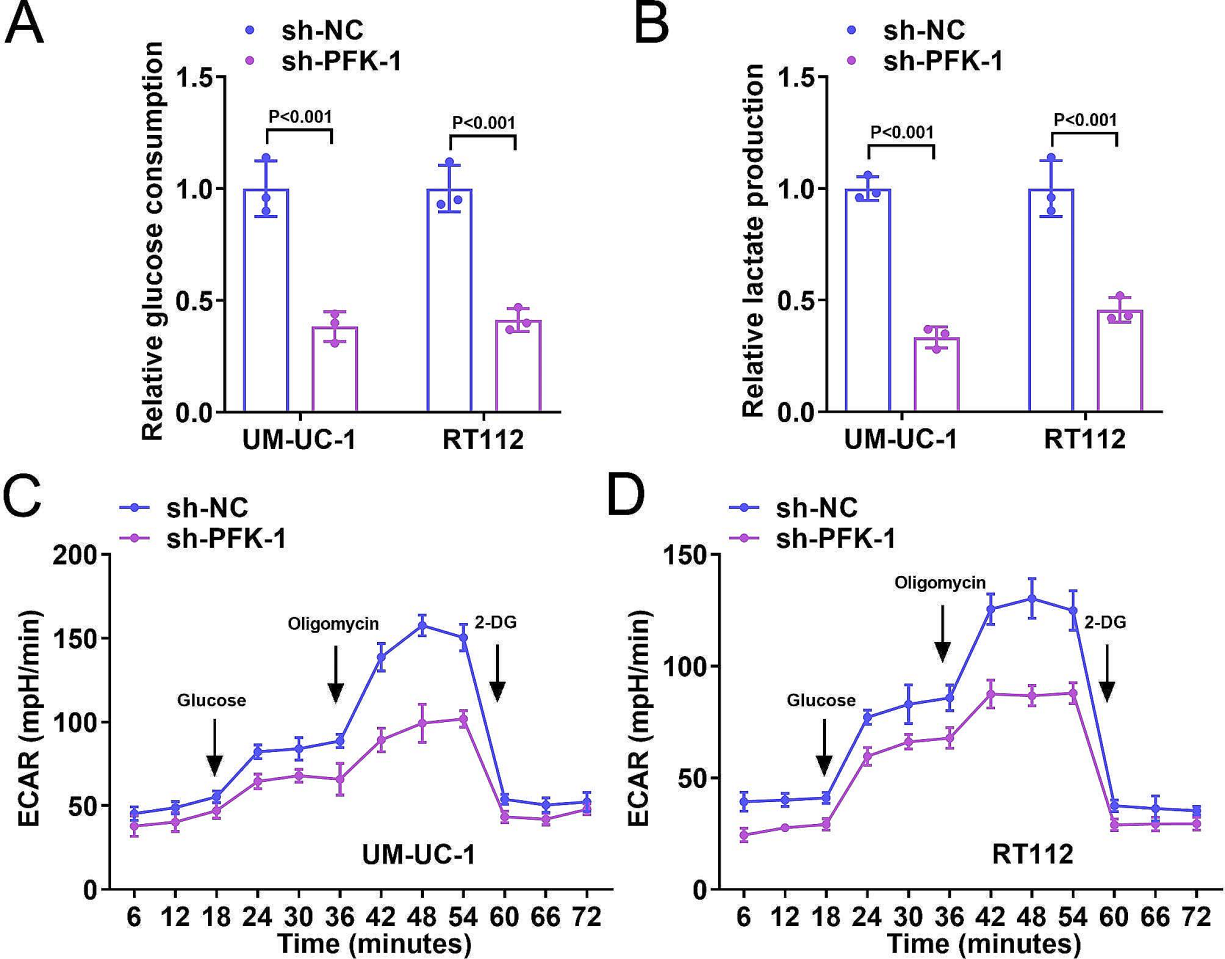
Knockdown of PFK-1 inhibited glycolysis of BC cells. After sh-PFK-1 transfection, relative glucose consumption (**A**), lactate production (**B**) and ECAR (**C**-**D**) were decreased in UM-UC-1 and RT112 cells

Subsequently, we investigated the role of PFK-1 in cellular glycolysis. We first investigated the effect of knockdown PFK-1 on the glucose metabolism in UM-UC-1 and RT112 cells. We observed that sh-PFK-1 transfection decreased the glucose consumption (Fig. 2A) and lactate production (Fig. 2B) levels. ECAR was significantly decreased in the sh-PFK-1 group compared to the sh-NC group (Fig. 2C, D). These results indicated that PFK-1 suppressed glycolysis of BC cells.

### PFK-1 suppressed the histone lactylation of BC cells and ZEB1 transcriptional activity

**Fig. 3:**
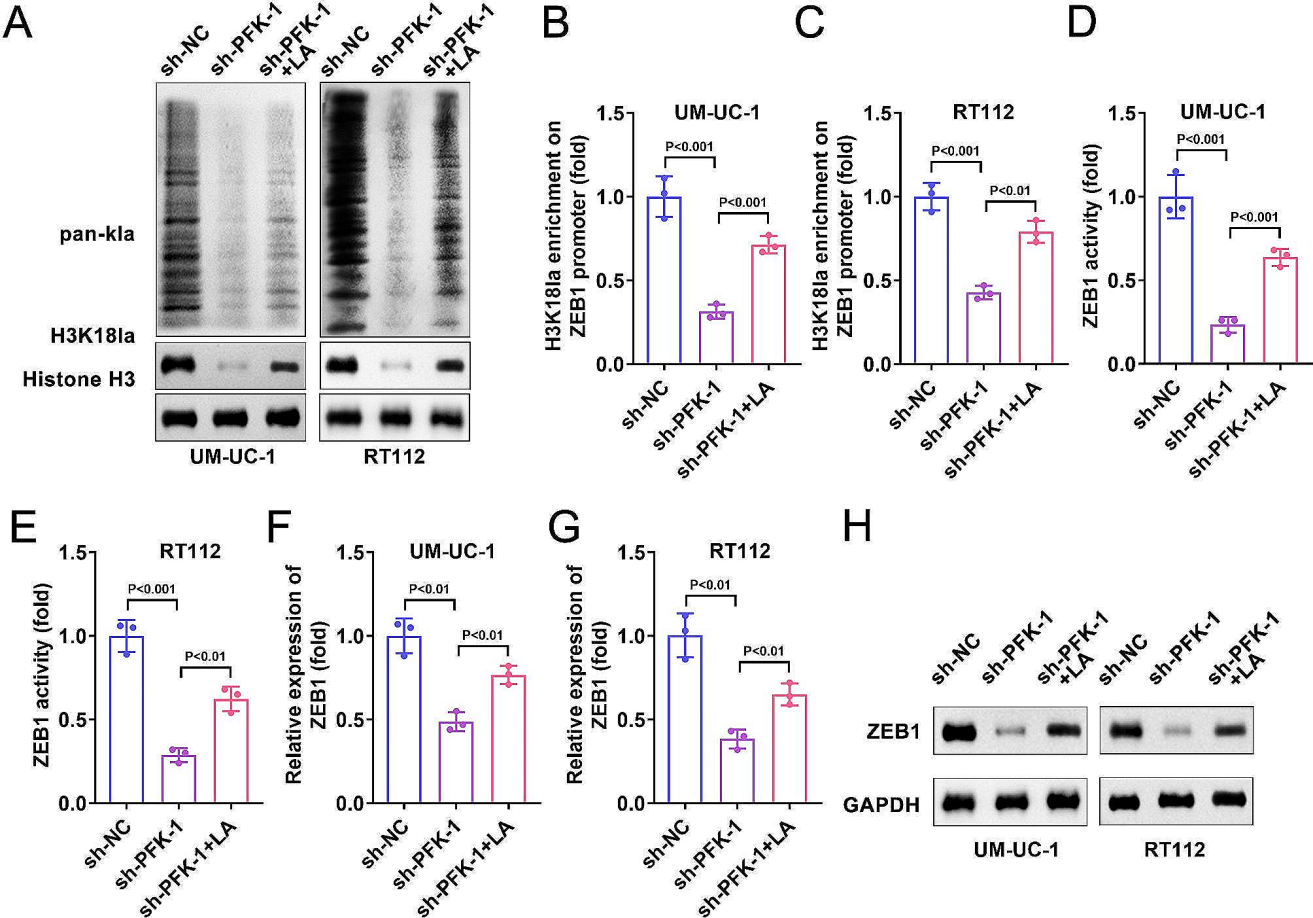
PFK-1 suppressed the histone lactylation of BC cells and ZEB1 transcriptional activity. After sh-PFK-1 transfection and LA treatment, (**A**) the expression of pan-kla and H3K18la was measured by western blot. (**B**, **C**) ChIP assay showed that PFK-1 knockdown inhibited the binding of H3K18 with the promoter of ZEB1. (**D**, **E**) Luciferase assay further indicated that PFK-1 knockdown suppressed the transcription activity of ZEB1. Sh-PFK-1 transfection decreased the mRNA (**F**-**G**) and protein (**H**) level of ZEB1ZEB1 mRNA expression in UM-UC-1 and RT112 cell lines

As PFK-1 is a critical metabolic enzyme in the glycolysis [15]. Lactate acid, which is the end-product of glycolysis has been confirmed to play crucial roles in the lactylation of proteins [16, 17]. Thus, we then evaluated the lactylation of BC cells. The results showed that total lactylation (pan-kla) and lactylation of histone (H3K18la) in the sh-PFK-1 group were both reduced compared with the sh-NC group (Fig. 3A). As reported, the lactylation of histone regulate the transcription of genes. In addition, ZEB1 was involved in proliferation, migration and glycolysis of cancer cells. Thus, we next checked whether histone lactylation modulate the transcription activity of ZEB1. As expected, ChIP assay showed that PFK-1 knockdown inhibited the binding of H3K18 with the promoter of ZEB1 in BC cells (Fig. 3B, C). Luciferase assay further indicated that PFK-1 knockdown suppressed the transcription activity of ZEB1 (Fig. 3D, E). Moreover, sh-PFK-1 was found to reduce the mRNA and protein level of ZEB1 (Fig. 3F-H). In addition, all these effect of PFK-1 silencing could be reversed by LA treatment. These results suggested that PFK-1 regulate the transcription activity of ZEB1 by modulating histone lactylation.

### ZEB1 overexpression reversed the effects of PFK-1 silencing on BC progression

**Fig. 4:**
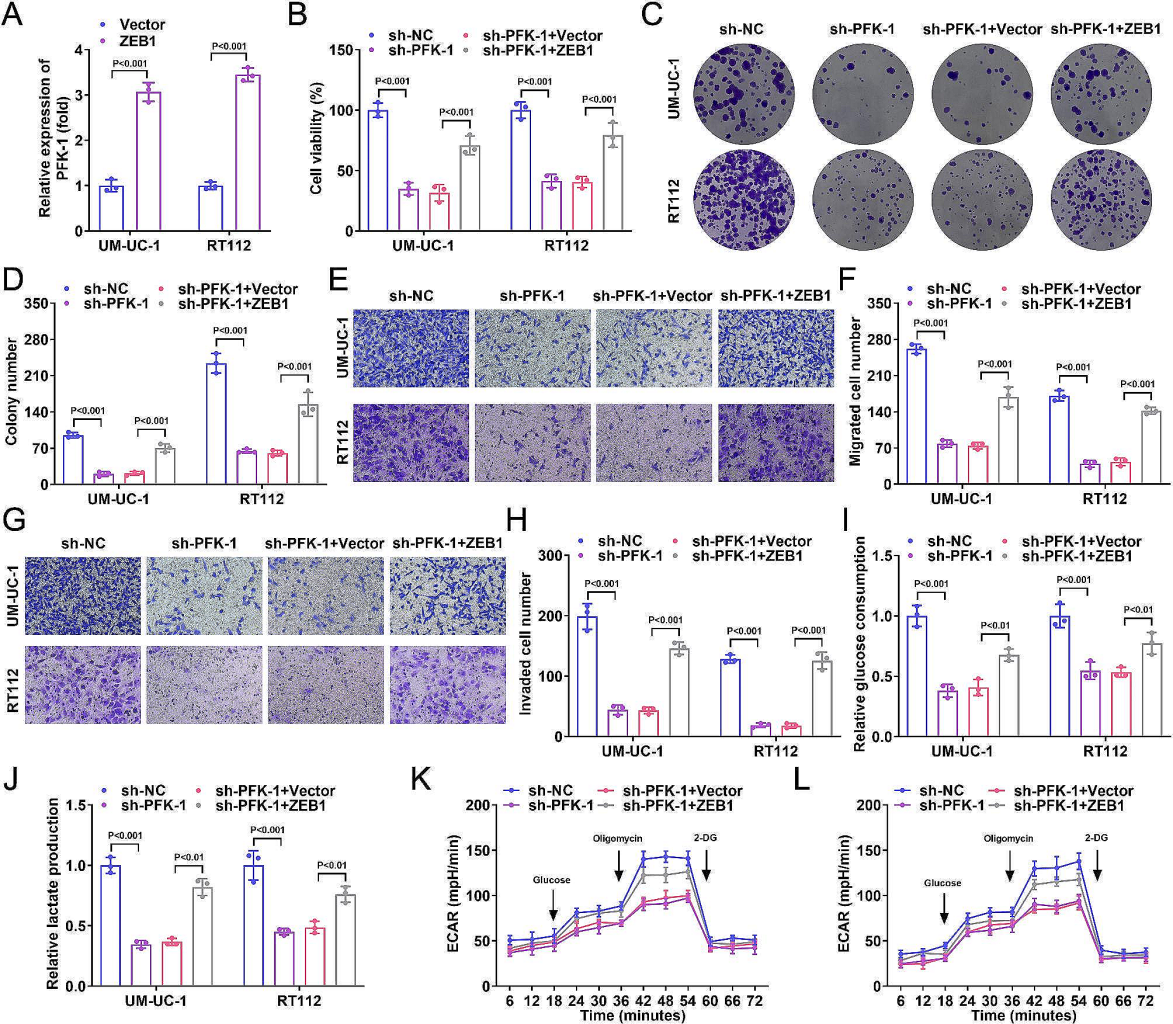
ZEB1 overexpression reversed the effects of PFK-1 silencing on BC progression. (**A**) After ZEB1 overexpressed vector transfection, the mRNA levels of ZEB1 were increased in UM-UC-1 and RT112 cells. The UM-UC-1 and RT112 cells were transfected with sh-PFK-1 and ZEB1 overexpressed vector. (**B**) CCK-8 assay showed ZEB1 overexpression increased the cell viability of UM-UC-1 and RT112 cells. (**C**-**D**) Colony formation assay showed ZEB1 overexpression increased cell growth of UM-UC-1 and RT112 cells. Transwell assay showed ZEB1 overexpression increased the migration (**E**-**F**) and invasion (**G**-**H**) of UM-UC-1 and RT112 cells. After ZEB1 overexpression, relative glucose consumption (**I**), lactate production (**J**) and ECAR (**K**-**L**) were increased in UM-UC-1 and RT112 cells

To investigate the role of ZEB1 in BC progression, we constructed a ZEB1 overexpression plasmid and transfected it into BC cell lines, which increased the ZEB1 levels in BC cell lines (Fig. 4A). We found that BC cell viability was reduced after transfection with sh-PFK-1, which was partially reversed by ZEB1 (Fig. 4B). In addition, cell proliferation (Fig. 4C, D), migration (Fig. 4E, F), invasion (Fig. 4G, H) abilities, glucose consumption (Fig. 4I), lactate production (Fig. 4J) and ECAR levels (Fig. 4K, L) were elevated in the sh-PFK-1 + ZEB1 group compared to the sh-PFK-1 + vector group. This suggests that ZEB1 overexpression could reverse the effects of PFK-1 silencing on BC progression in vitro.

### Knockdown of PFK-1 alleviated BC cancer progression in vitro

**Fig. 5:**
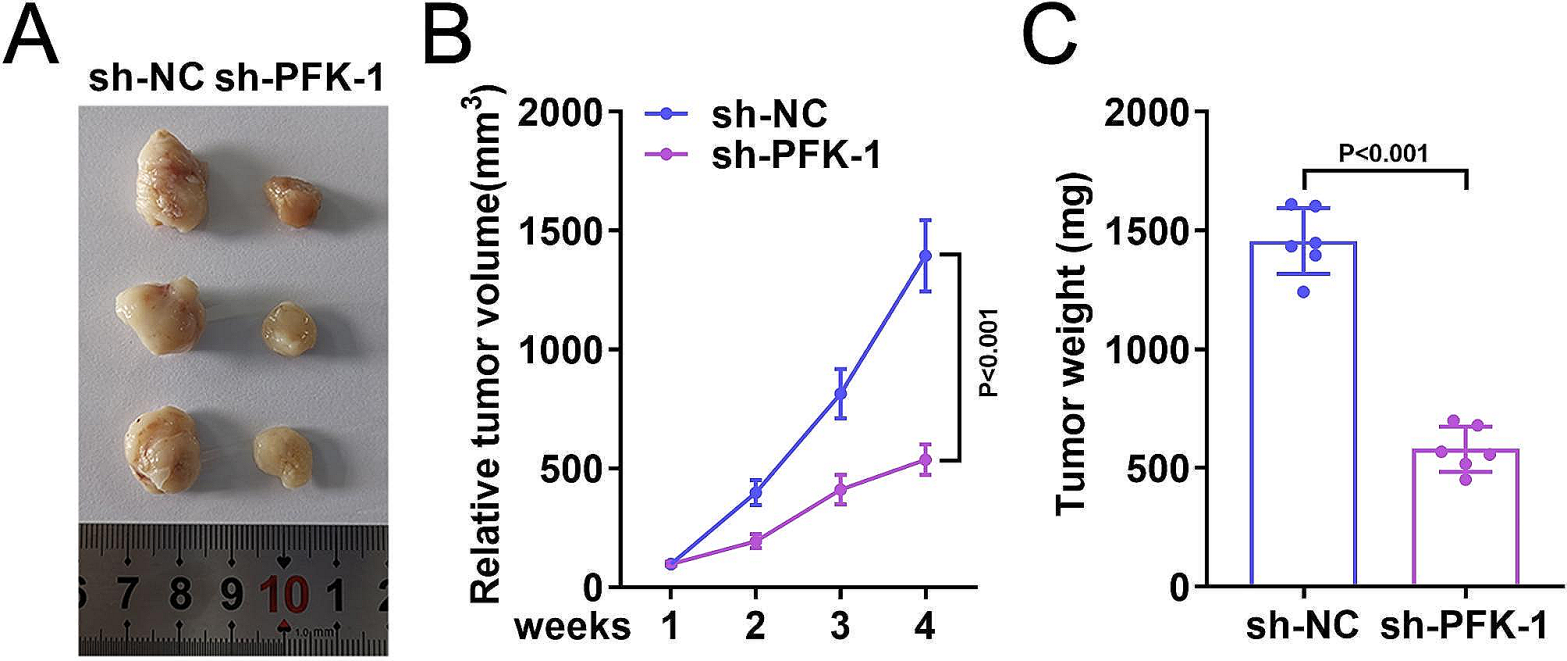
Knockdown of PFK-1 inhibits the growth of BC cells in nude mice. (**A**) UM-UC-1 cells transfected with shNC or shPFK-1 were injected into BALB/C nude mice. The volume (**B**) and weight (**C**) of tumor in shPFK-1 transfection nude mice were decreased

Finally, the effect of PFK-1 in vivo was verified. The results revealed that the tumor volume were significantly decreased in sh-PFK-1 group compared to the sh-NC group (Fig. 5A-B). In addition, the weight of tumor in the sh-NC group was significantly higher than that in the sh-PFK-1 group (Fig. 5C).

## Discussion

In this study, we demonstrated that PFK-1 silencing inhibits the proliferation, migration, invasion and glycolysis of BC cells. Mechanistically, PFK-1 promotes the histone lactylation and the transcriptional activity of ZEB1.

Aerobic glycolysis, also known as the Warburg effect, is one of the most important mechanisms in tumourigenesis and progression [18–21]. During glycolysis, tumour cells consume glucose, which produces lactic acid and released into the tumour micro-environment (TME), lowering the pH of the TME [22].

Numerous studies have shown that inhibition of glycolysis improves bladder tumours. Some studies have shown that melatonin inhibits bladder tumourigenesis by inhibiting glycolysis [23, 24]. HNRNPL increases bladder cancer immunotherapy sensitivity by inhibiting glycolysis [21]. Here, we found that PFK-1 knockdown inhibited glycolysis of BC cells which is consistent with the previous studies. As previously mentioned, PFK-1 catalyses the conversion of FBPase, which induces the tetramerisation of pyruvate kinase (PK) into the glycolytic enzyme complex, thereby promoting the glycolytic pathway. We verified that PFK-1 knockdown reduced the glycolysis level of BC cells. However, the precise mechanism remain unclear.

Recently, lactylation, as a novel identified post-transcriptional modification, has been determined to play critical roles in regulating functions of protein and thus participate in cell process and development of disease. As we know, lactate is the end product of glycolysis. Since the Warburg effect (aerobic glycolysis) is one of the hallmarks of cancer, even under aerobic conditions, cancer cells tend to “ferment” glucose to lactate to produce energy, and metabolize more lactate from glucose than normal cells in a given period of time [25]. Therefore, histone lactylation in tumours is likely to be aberrant and it is essential to explore the potential function of histone lactylation in tumourigenesis. It is shown that exogenous and endogenous lactate directly promotes lysine-lactoylation, that inhibitors of lactate-depleted glycolysis decrease lysine-lactoylation, and that mitochondrial inhibitors or hypoxia with increased lactate production amplify lysine-lactoylation [14].

We speculated whether PFK-1 could change the lactylation of BC cells. The results confirmed our speculation. PFK-1 silencing notably reduced the total lactylation of BC cells. It has been widely studied and confirmed that histone lactylation involves in the tanscriptional activation of downstream genes.

Zinc finger E-box-binding homeobox 1 (ZEB1) is a member of the ZEB family, which contains two zinc finger clusters at the N- and C-terminus, respectively, that bind to the E2 box-like CACCT(G) sequence in the promoter region of target genes [26]. ZEB1 is highly expressed in cancers such as colorectal, pancreatic, oesophageal squamous cell and bladder cancer and contributes to the progression of these tumours [27]. For instance, ZEB1 promotes the progression of BC by promoting biogenesis of circNIPBL and activating Wnt/β-catenin pathway [13]. This study demonstrated that PFK-1 knockdown suppressed the histone lactylation. Histone lactylation has been confirmed to regulate cell biological functions by activating downstream gene transcription and expression [28, 29]. As ZEB1 involves in proliferation, metastasis and glycolysis of cancer cells, we tried to find out whether PFK-1 could modulate the expression of ZEB1. Through the ChIP assay, we found that PFK-1 knockdown inhibited the binding of H3K18 with the promoter of ZEB1. The Luciferase assay further indicated that PFK-1 knockdown suppressed the transcription activity of ZEB1. These results preliminarily indicated that PFK-1 regulated activity of ZEB1 through histone lactylation mediated transcription regulation. However, the regulation mechanism between H3K181a and PFK-1 remains further experiments to clarify.

In spite of histone lactylation, it has been found that lactylation of other proteins regulate their functions. For instance, PKM2 lactylation inhibits inflammatory metabolic adaptation in macrophages. Lactate promotes macrophage HMGB1 lactylation, acetylation, and exosomal release in polymicrobial sepsis [30–32]. Whether PFK-1 could modulate the lactylation of ZEB1 will be studied in our further research work.

In conclusion, we have identified a novel mechanism by which PFK-1 inhibits BC by inhibiting ZEB1 to reduce lactate modification, establishing a direct link between PFK-1 and ZEB1 inhibition of glycolysis. Therefore, regulating the PFK-1/ZEB1 axis to inhibit the malignant behavior of BC cells may be a promising approach for future BC treatment. Furthermore, more animal experiments and clinical studies are needed to further confirm this viewpoint.

### Electronic supplementary material

Below is the link to the electronic supplementary material.

Supplementary Material 1

Supplementary Material 2

## Data Availability

The datasets used and/or analyzed during the current study are available from the corresponding author on reasonable request.
