# Supplementary material for: The mechanism of PFK-1 in the occurrence and development of bladder cancer by regulating ZEB1 lactylation
Source: BMC Urol. 2024 Mar 13;24:59. doi: 10.1186/s12894-024-01444-5 (PMC10935987; doi:10.1186/s12894-024-01444-5)
Supplement: Supplementary file 1 — Supplementary Material 1 [file 12894_2024_1444_MOESM1_ESM.docx]

Fig.1D

|  | sh-NC | sh-PFK-1 |
| --- | --- | --- |
| UM-UC-1 | 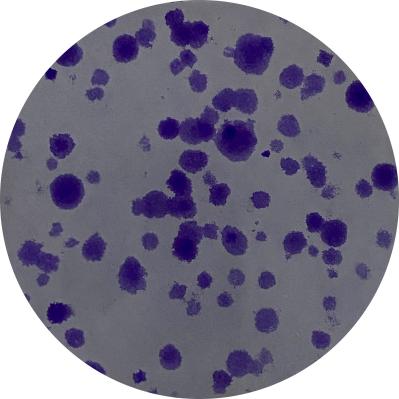 | 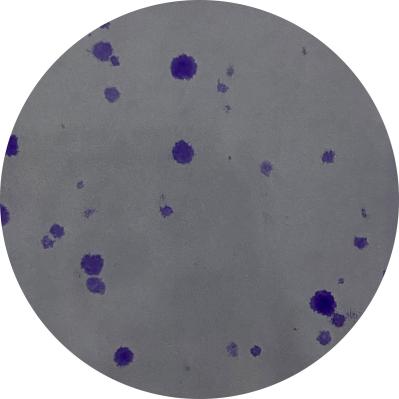 |
| RT112 | 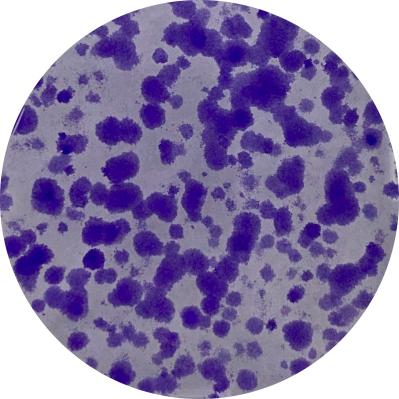 | 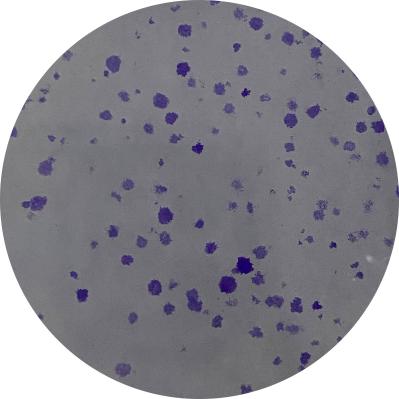 |

Fig.1F

|  | sh-NC | sh-PFK-1 |
| --- | --- | --- |
| UM-UC-1 | 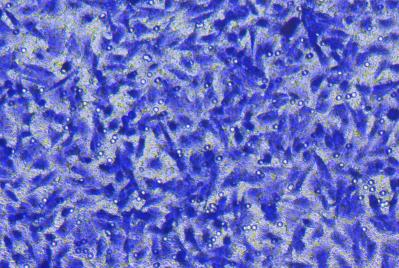 | 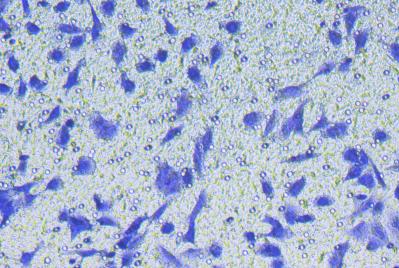 |
| RT112 | 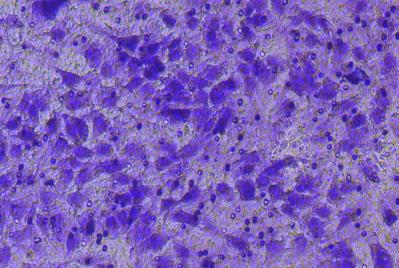 | 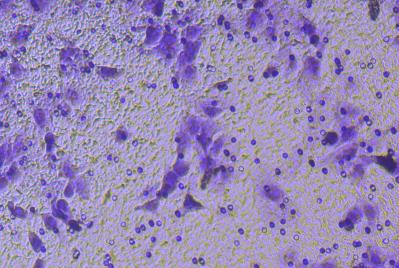 |

Fig.1H

|  | sh-NC | sh-PFK-1 |
| --- | --- | --- |
| UM-UC-1 | 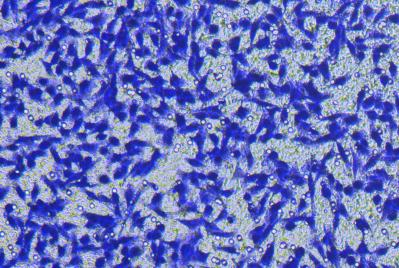 | 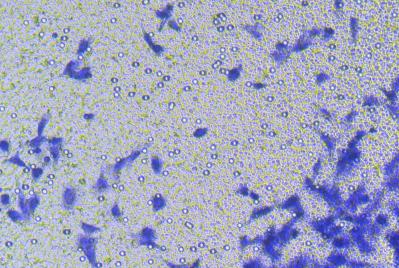 |
| RT112 | 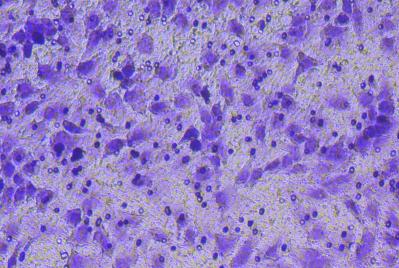 | 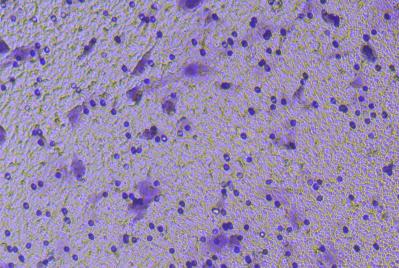 |

Fig.3A

|  | sh-NC | sh-PFK-1 | sh-PFK-1+LA | sh-NC | sh-PFK-1 | sh-PFK-1+LA |
| --- | --- | --- | --- | --- | --- | --- |
| pan-kla | 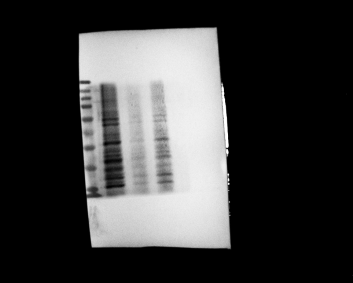 | | | 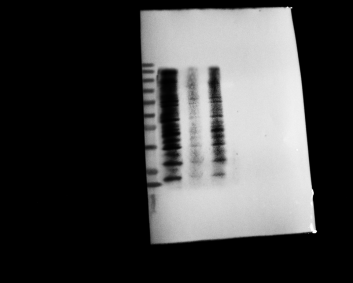 | | |
| H3K18la | 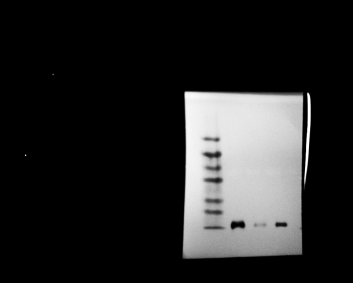 | | | 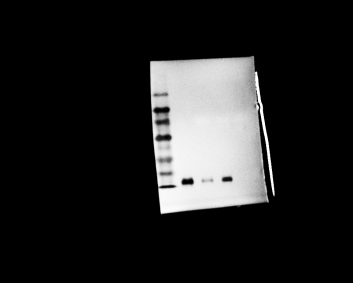 | | |
| Histone H3 | 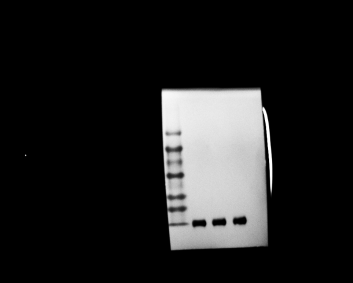 | | | 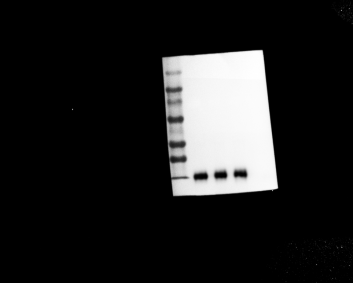 | | |

Fig.3H

|  | sh-NC | sh-PFK-1 | sh-PFK-1+LA | sh-NC | sh-PFK-1 | sh-PFK-1+LA |
| --- | --- | --- | --- | --- | --- | --- |
| ZEB1 | 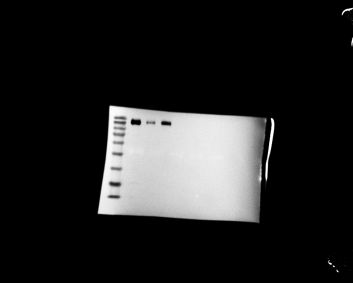 | | | 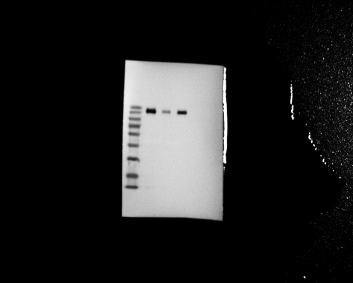 | | |
| GAPDH | 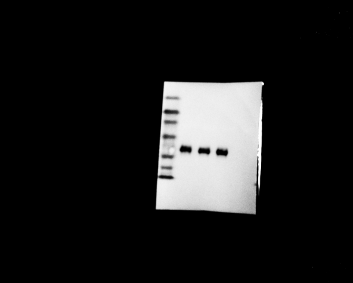 | | | 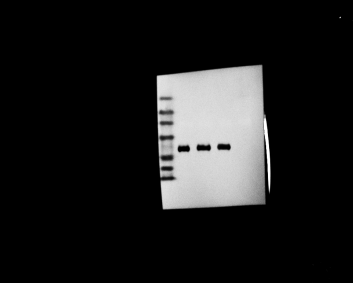 | | |

Fig.4C

|  | sh-NC | sh-PFK-1 | sh-PFK-1+Vector | sh-PFK-1+ZEB1 |
| --- | --- | --- | --- | --- |
| UM-UC-1 | 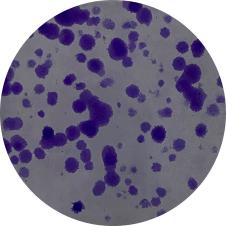 | 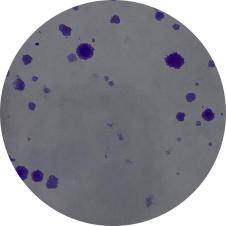 | 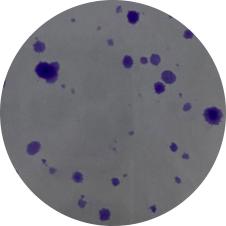 | 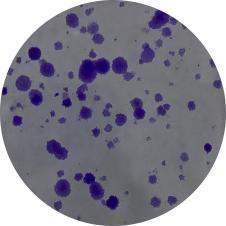 |
| RT112 | 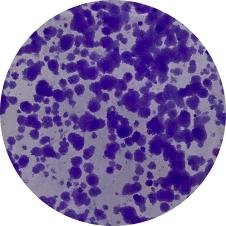 | 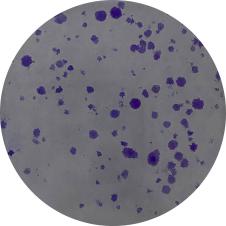 | 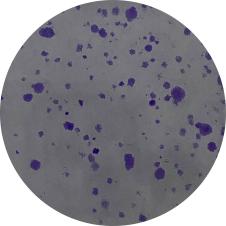 | 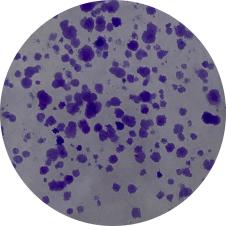 |

Fig.4E

|  | sh-NC | sh-PFK-1 | sh-PFK-1+Vector | sh-PFK-1+ZEB1 |
| --- | --- | --- | --- | --- |
| UM-UC-1 | 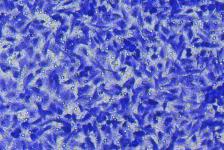 | 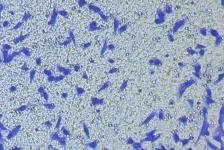 | 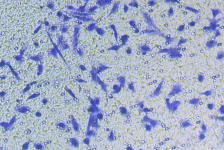 | 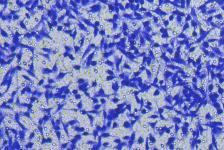 |
| RT112 | 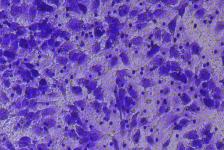 | 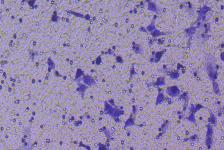 | 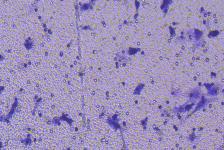 | 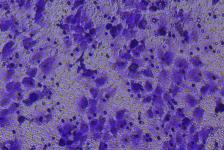 |

Fig.4G

|  | sh-NC | sh-PFK-1 | sh-PFK-1+Vector | sh-PFK-1+ZEB1 |
| --- | --- | --- | --- | --- |
| UM-UC-1 | 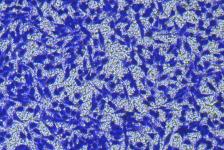 | 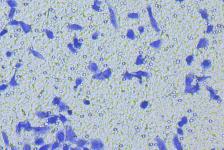 | 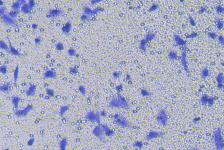 | 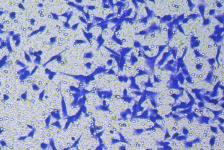 |
| RT112 | 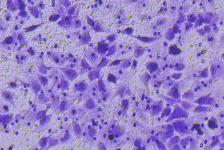 | 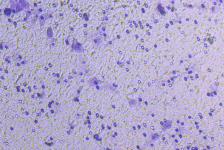 | 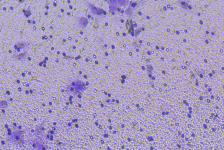 | 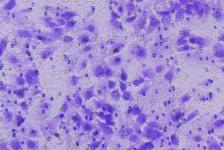 |

Fig.5A

| sh-NC | sh-PFK-1 |
| --- | --- |
| 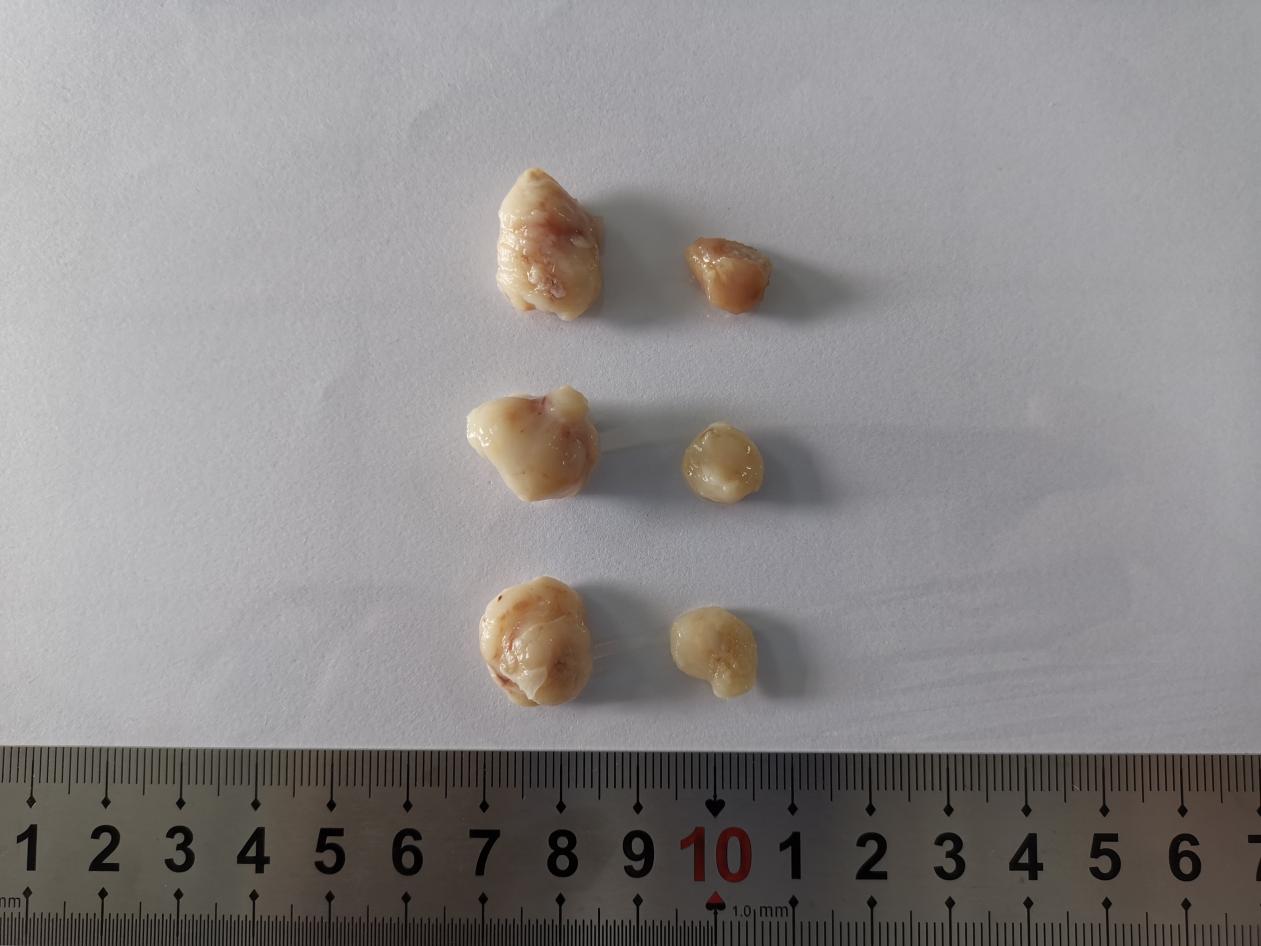 | |
